# Author Correction: Surfaceome dynamics reveal proteostasis-independent reorganization of neuronal surface proteins during development and synaptic plasticity

**DOI:** 10.1038/s41467-020-19654-4

**Published:** 2020-11-06

**Authors:** Marc van Oostrum, Benjamin Campbell, Charlotte Seng, Maik Müller, Susanne tom Dieck, Jacqueline Hammer, Patrick G. A. Pedrioli, Csaba Földy, Shiva K. Tyagarajan, Bernd Wollscheid

**Affiliations:** 1 Neuroscience Center Zurich, Zurich, Switzerland; 2 Institute of Translational Medicine (ITM), Department of Health Sciences and Technology, ETH Zurich, 8093 Zurich, Switzerland; 3 Swiss Institute of Bioinformatics (SIB), Lausanne, Switzerland; 4 Institute of Pharmacology and Toxicology, University of Zurich, Zurich, Switzerland; 5 Laboratory of Neural Connectivity, Faculties of Medicine and Natural Sciences, Brain Research Institute, University of Zurich, Zürich, 8057 Switzerland; 6 Max Planck Institute for Brain Research, Frankfurt, Germany

**Keywords:** Protein-protein interaction networks, Mass spectrometry, Neuronal development, Synaptic plasticity

Correction to: *Nature Communications* 10.1038/s41467-020-18494-6, published online 5 October 2020.

The original version of this Article contained errors in Figs. 3b, c and 6h in which the edges connecting the network nodes have been missing. This has been corrected in both the PDF and HTML versions of the Article.

