# Supplementary material for: Surfaceome dynamics reveal proteostasis-independent reorganization of neuronal surface proteins during development and synaptic plasticity
Source: Nat Commun. 2020 Oct 5;11:4990. doi: 10.1038/s41467-020-18494-6 (PMC7536423; doi:10.1038/s41467-020-18494-6)
Supplement: Supplementary file 1 — Supplementary Information [file 41467_2020_18494_MOESM1_ESM.pdf]

Surfaceome dynamics during neuronal development and synaptic plasticity reveal system-wide surfaceome reorganization independent of global proteostasis

van Oostrum et al.

Supplementary Information

Supplementary Discussion

Here, we present an initial blueprint of how many, and which, proteins are present on the surface of neurons. First, the autoCSC workflow includes peptide enrichment and probabilistic identification by mass spectrometry, producing false positives and bias in cell surface protein identifications. Second, neurons come in different populations and our rat cortical neuronal culture system contains a mixture of these cells. Furthermore, our cultures contain predominantly neurons, but also astrocytes. We addressed this question partially by use of transcriptomic data; a more complete delineation could be achieved by combination with cell-type specific metabolic labeling approaches[1,2]. From a methodological point of view, there are two major biases in our analysis. First, our approach is limited to proteins with extracellular *N*-glycosylation. It is well established that *N*-glycosylation is a common feature among surface proteins[3] and more than 95% of cell surface proteins have an extracellular glycosylation motif[4]. Nevertheless, there are proteins with important surface functions, for example AMPA auxiliary subunits[5,6], that are not glycosylated and thus were not considered here. More crucially, we only identified *N*-glycosylated sites if they were present within a tryptic peptide that is proteotypic (after protein grouping) and suitable for MS analysis, which depends on biophysical properties such as peptide length. Although we calculated that a large proportion of *N*-glycosylation sites are located within proteotypic peptides after tryptic digestion (>90% for mouse and human *N*-glycosylation sites in Uniprot), the percentage of resulting peptides that are detected by MS is more difficult to assess. We can grossly approximate the number of missed *N*-glycoproteins by extrapolation from the percentage of missed additional glycosylation sites for proteins with confirmed presence by autoCSC. For a comparable autoCSC dataset with human cells[7], we calculated the percentage of missed glycosylation sites (retrieved from Uniprot) in the range of two-thirds of all potentially present *N*-glycosites. Assuming two *N*-glycosites per surface protein on average, we estimate that about one-third of surface proteins were not detected. This suggests an approximate size of 1500 proteins for the cortical neuronal culture surfaceome. We provide experimental evidence for 1,786 unique extracellular *N*-glycosylation sites. Of these, as shown by comparison with Uniprot, 1,345 were not previously described. It should be noted that annotation for rat proteins is considerably less comprehensive than for human or mouse.

Supplementary References

1. Alvarez-Castelao, B. *et al.* Cell-type-specific metabolic labeling of nascent proteomes in vivo. *Nat. Biotechnol.* **35**, 1196–1201 (2017).
2. Mahdavi, A. *et al.* Engineered Aminoacyl-tRNA Synthetase for Cell-Selective Analysis of Mammalian Protein Synthesis. *J. Am. Chem. Soc.* **138**, 4278–4281 (2016).
3. Moremen, K. W., Tiemeyer, M. & Nairn, A. V. Vertebrate protein glycosylation: diversity, synthesis and function. *Nat. Rev. Mol. Cell Biol.* **13**, 448–462 (2012).

4. Bausch-Fluck, D. *et al.* The in silico human surfaceome. *Proceedings of the National Academy of Sciences* 201808790 (2018).

5. Schwenk, J. *et al.* Regional Diversity and Developmental Dynamics of the AMPA-Receptor Proteome in the Mammalian Brain. *Neuron* **84**, 41–54 (2014).

6. Schwenk, J. *et al.* Functional proteomics identify cornichon proteins as auxiliary subunits of AMPA receptors. *Science* **323**, 1313–1319 (2009).

7. van Oostrum, M. *et al.* Classification of mouse B cell types using surfaceome proteotype maps. *Nat. Commun.* **10**, 5734 (2019).

Supplementary Figure 1

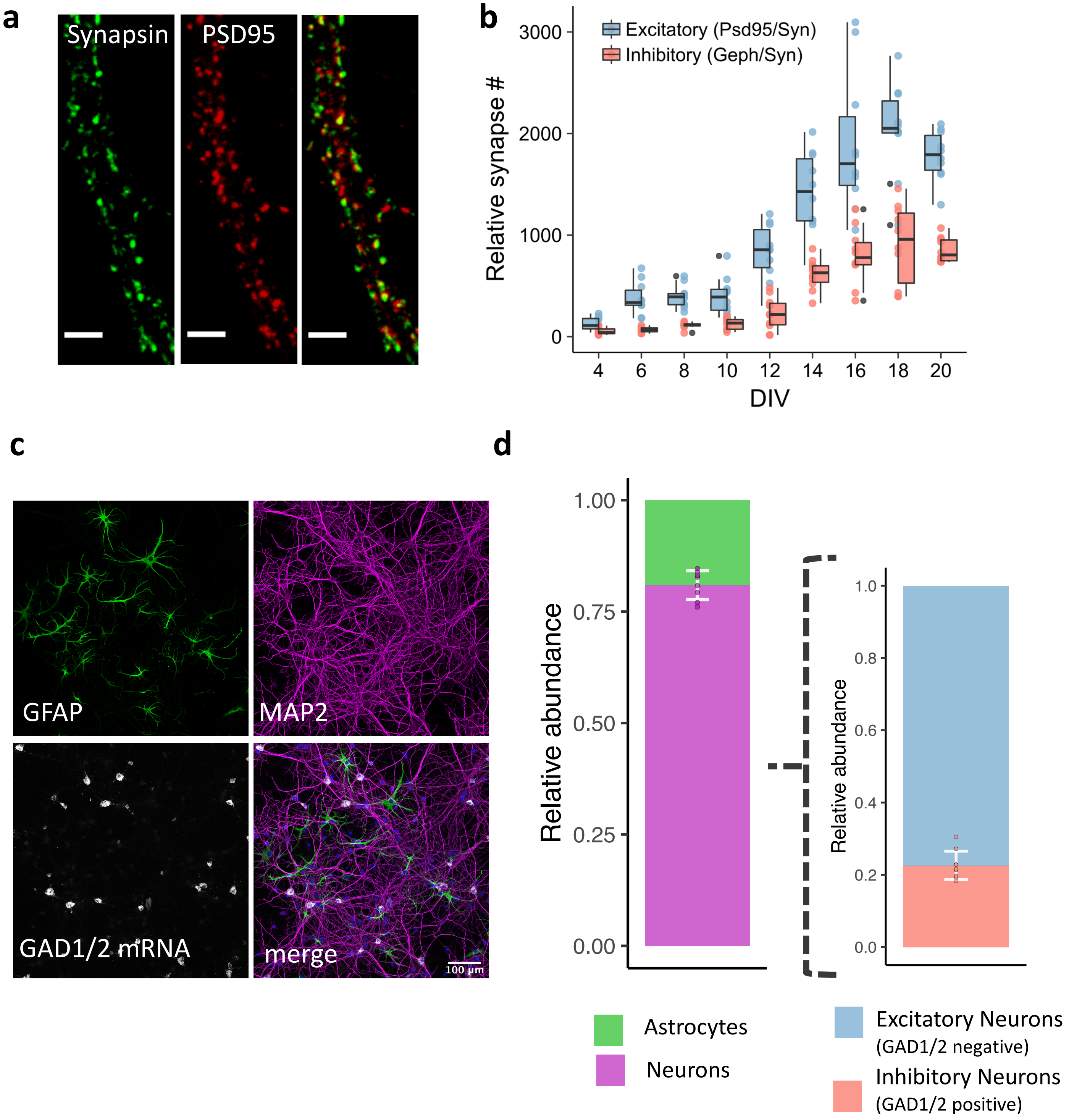

Supplementary Figure 1. a Representative image of a mature neuronal process used for synapse number quantification (DIV20). Bars 3 µm. **b** Relative number of synapses (determined by counting puncta stained with Psd95 and Synapsin or Gephyrin and Synapsin) as a function of time during neurodevelopment in cortical cultures. Boxes indicate median and percentiles (25th and 75th). **c** Representative overview image of a cortical neuron culture with astrocytes (GFAP), neurons (MAP2) and inhibitory neurons (GAD1/2 mRNA) visualized (DIV12). Bar 100 µm. **d** Relative abundance of astrocytes, neurons and inhibitory neurons in cortical cultures. Error bars indicate average and standard deviation.

Supplementary Figure 2

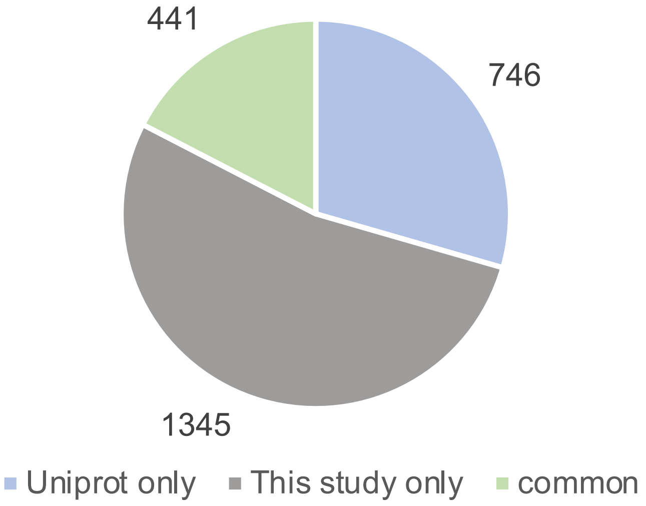

Supplementary Figure 2. Overview of unique *N*-glycosylation sites. Pie chart illustrating overlap of quantified glycosylation sites identified in this study with the UniProt database of quantified proteins. In the Uniprot reference proteome of *Rattus norvegicus*, 73% of proteins are not reviewed and do not contain any annotation on glycosylation sites.

Supplementary Figure 3

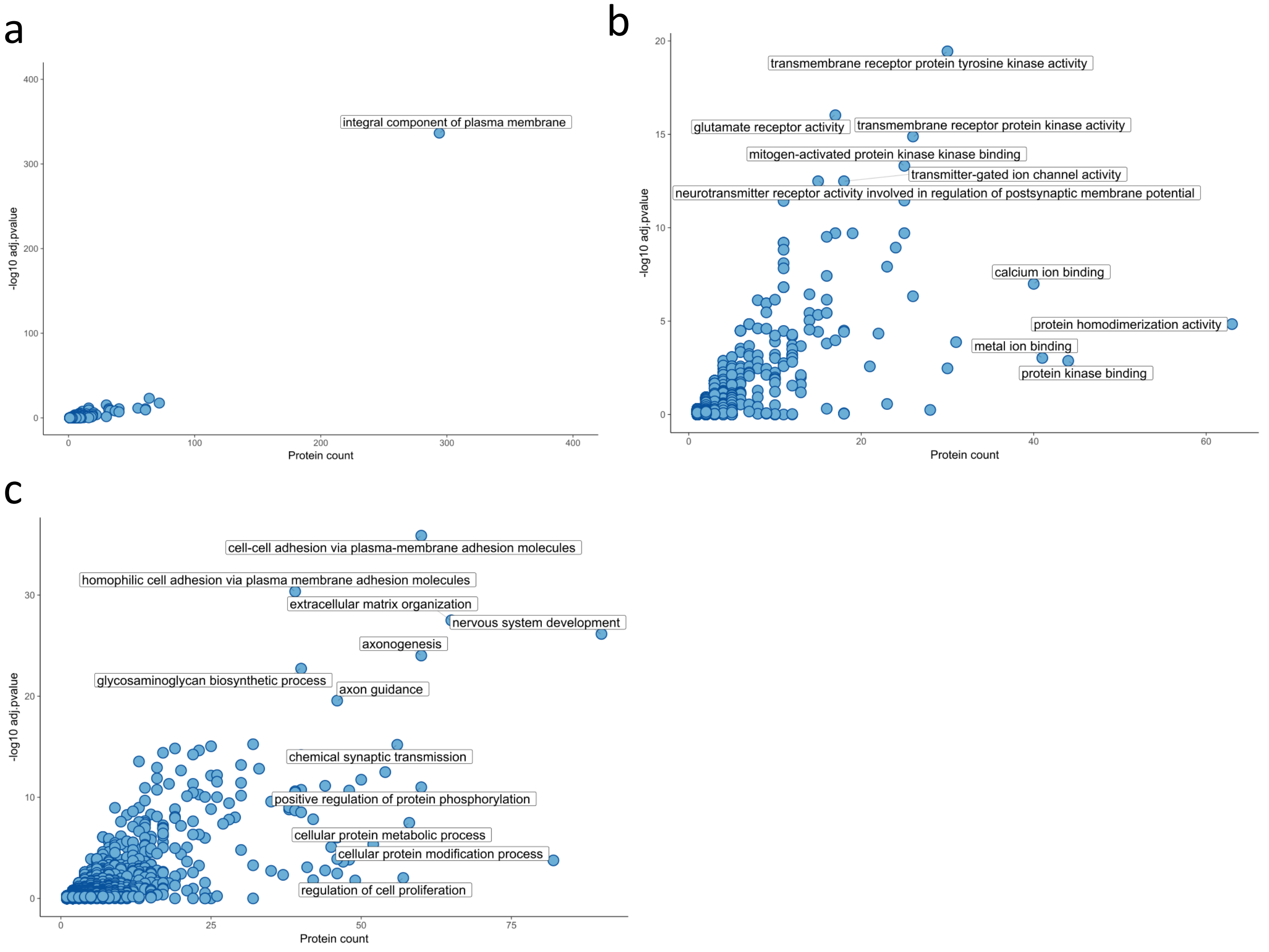

Supplementary Figure 3. Enrichment of GO terms for the neuronal surfaceome. Significantly enriched **a** cellular components, **b** molecular functions, and **c** biological processes for the quantified neuronal surfaceome ($p < 0.05$, Fisher's exact test) plotted against the protein count for each term.

Supplementary Figure 4

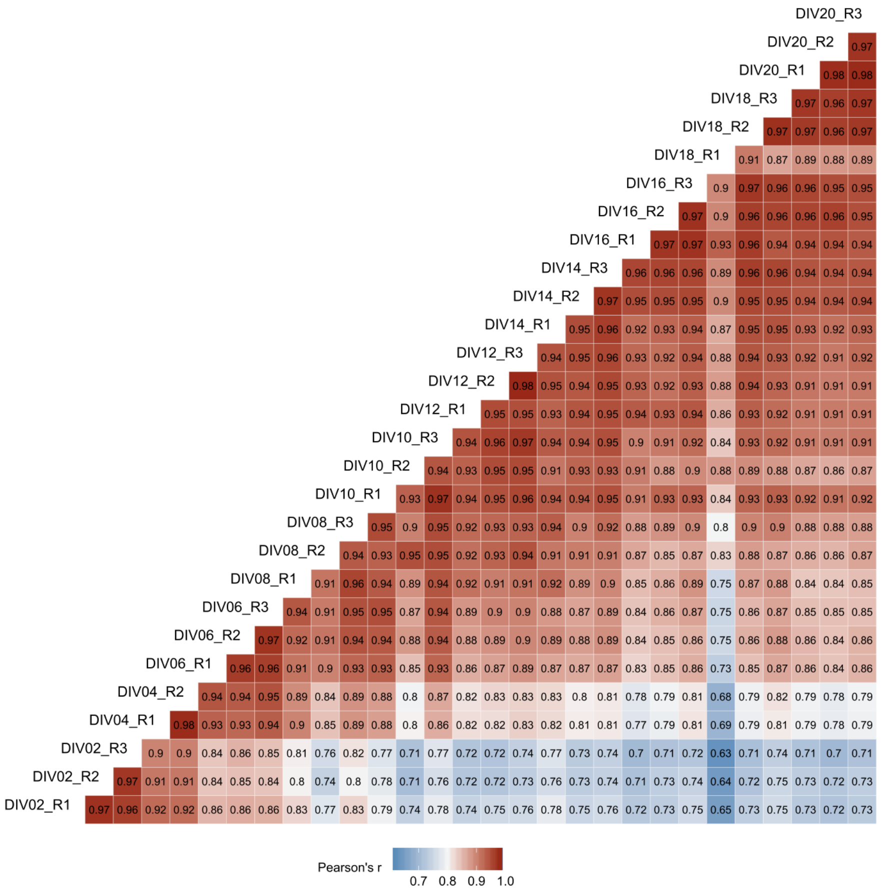

Supplementary Figure 4. Correlation matrix of all samples and developmental time points. Pearson's correlation of all quantified protein abundance values per DIV for all replicates.

Supplementary Figure 5

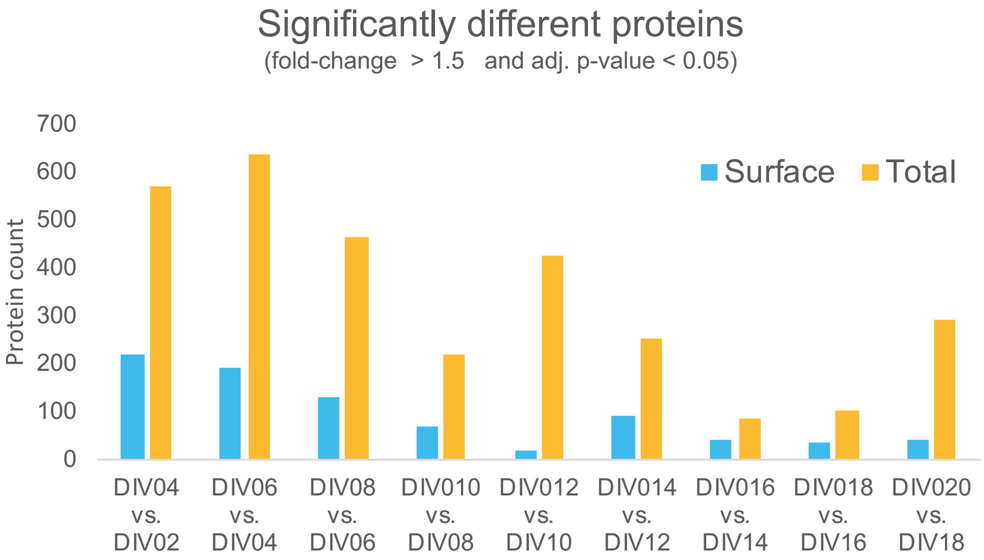

Supplementary Figure 5. Counts of significantly different proteins for neighboring time points. Result of pairwise significance testing for surface and total abundance across the developmental time series. Quantitative values obtained from MSstats.

Supplementary Figure 6

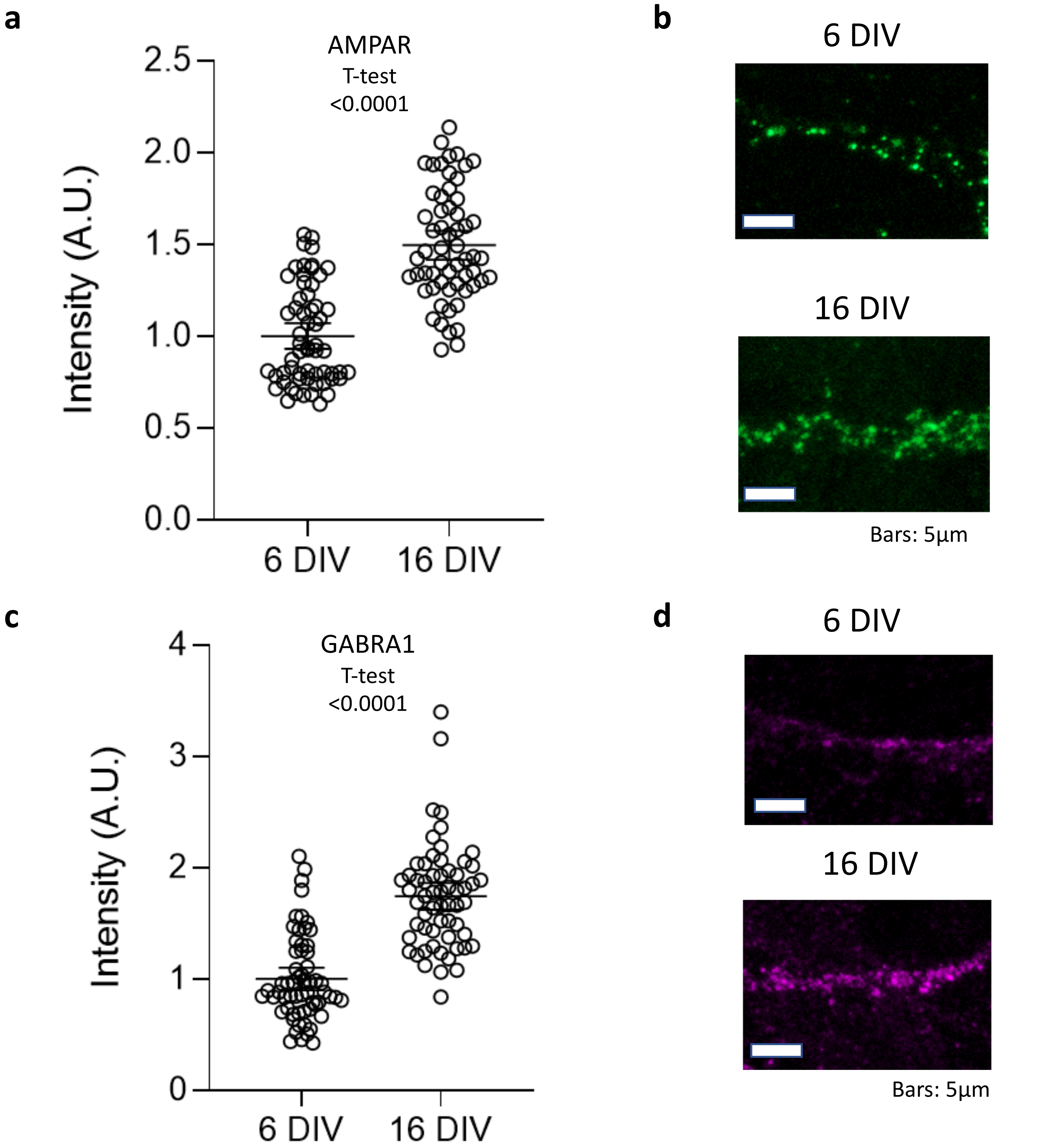

Supplementary Figure 6. a Quantification of mean fluorescent intensity of antibody signal on the primary dendrite surface using antibodies against GluA. GluA: n = 60 (DIV6), n = 59 (DIV16). **b** Representative images of analysed primary dendrites stained for GluA at DIV 6 and DIV16. Bars 5 µm. **c** Quantification of mean fluorescent intensity of antibody signal on the primary dendrite surface using antibodies against GABRA1. GABRA1: n = 60 (DIV6 and DIV16). **d** Representative images of analysed primary dendrites stained for GABRA1 at DIV 6 and DIV16. Bars 5 µm. Means + 95% CI presented.

Supplementary Figure 7

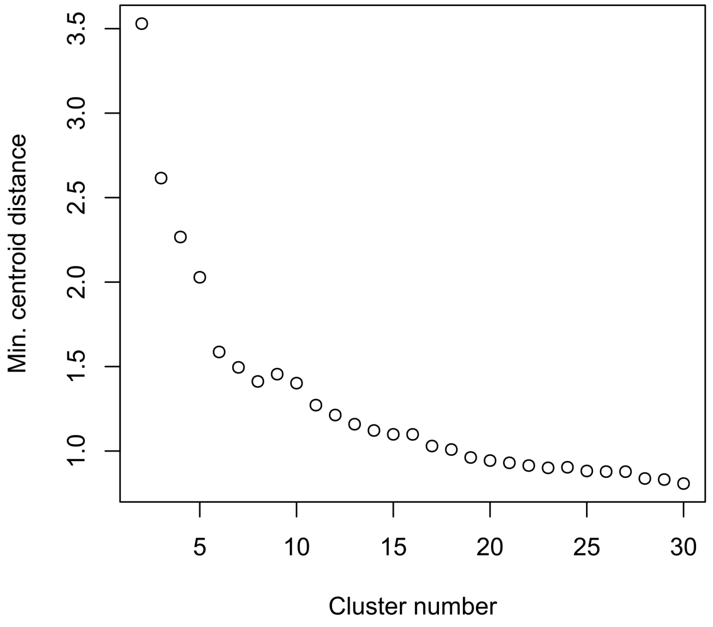

Supplementary Figure 7. Minimum centroid distance plot for determination of optimal cluster number. Calculation of minimum centroid distance for a range of cluster numbers used to estimate the optimal number of clusters as previously described (Kumar, Lokesh, and Matthias E Futschik. 2007).

Supplementary Figure 8

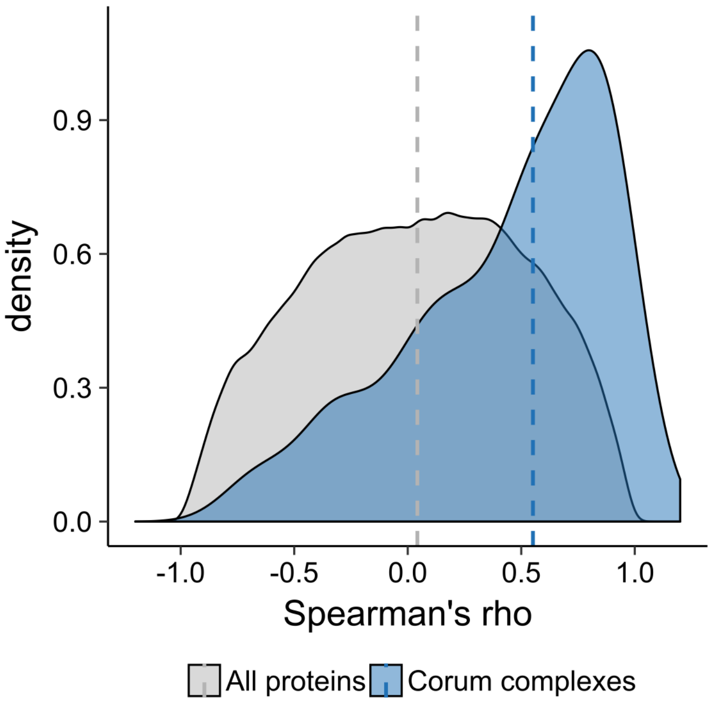

Supplementary Figure 8. Correlation of surface abundance profiles with membership in protein complexes. Density plot of Spearman's correlation coefficients for proteins with a complex member (Corum database, median = 0.55) or all protein combinations (median 0.04) ($p = 1.095e^{-07}$, t-test).

Supplementary Figure 9

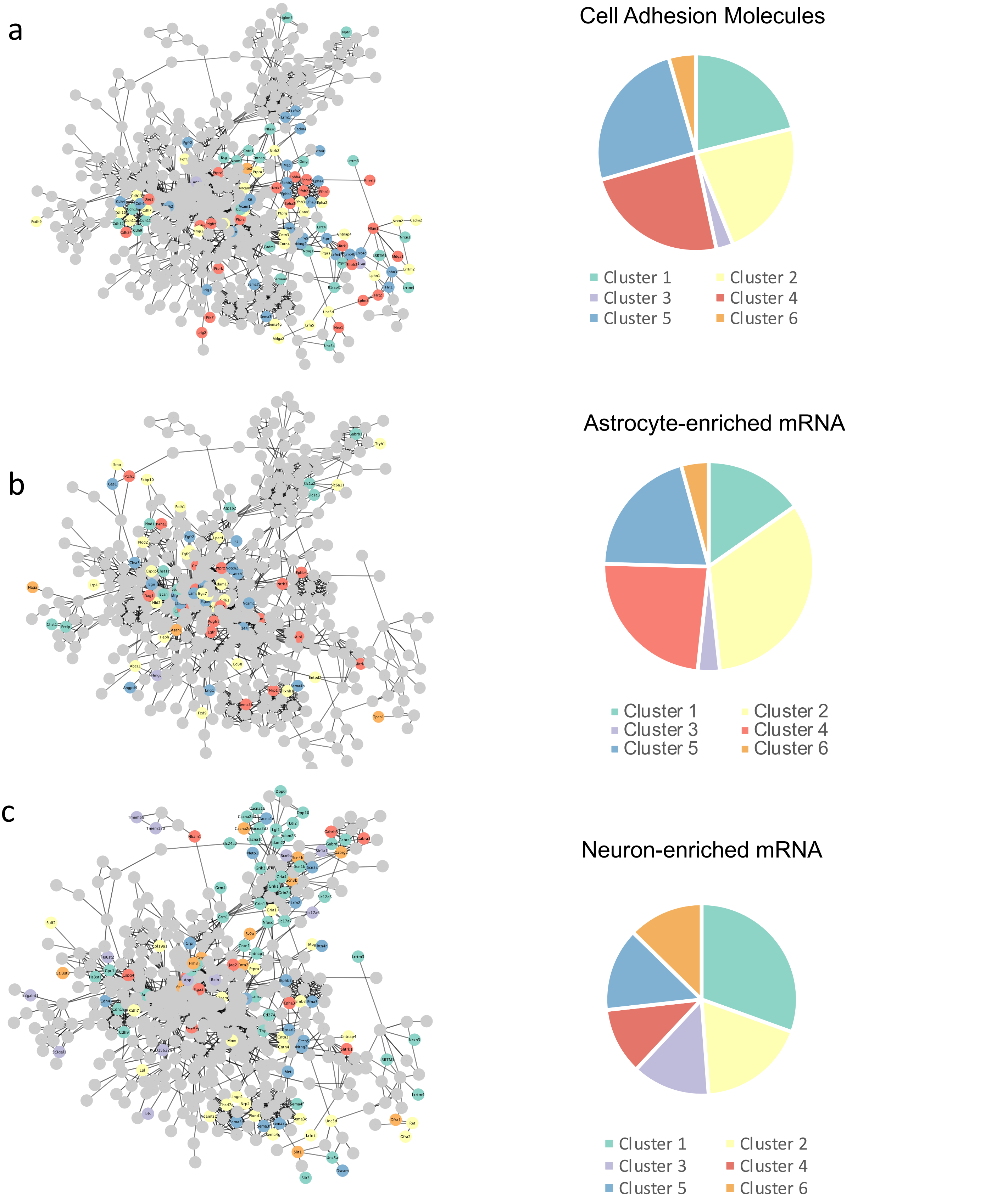

Supplementary Figure 9. Defined protein categories mapped to the neuronal surfaceome string network. a Synaptic cell adhesion molecules as defined in Földy et al.[46] mapped to the surfaceome network. **b** and **c** Proteins encoded by mRNAs enriched in **b** astrocytes or **c** neurons[48] mapped to the largest connected cluster of quantified surface proteins within the string network representation. Edges represent string confidence of 0.7. Pie charts illustrate the proportion of proteins that belong to each cluster.

Supplementary Figure 10

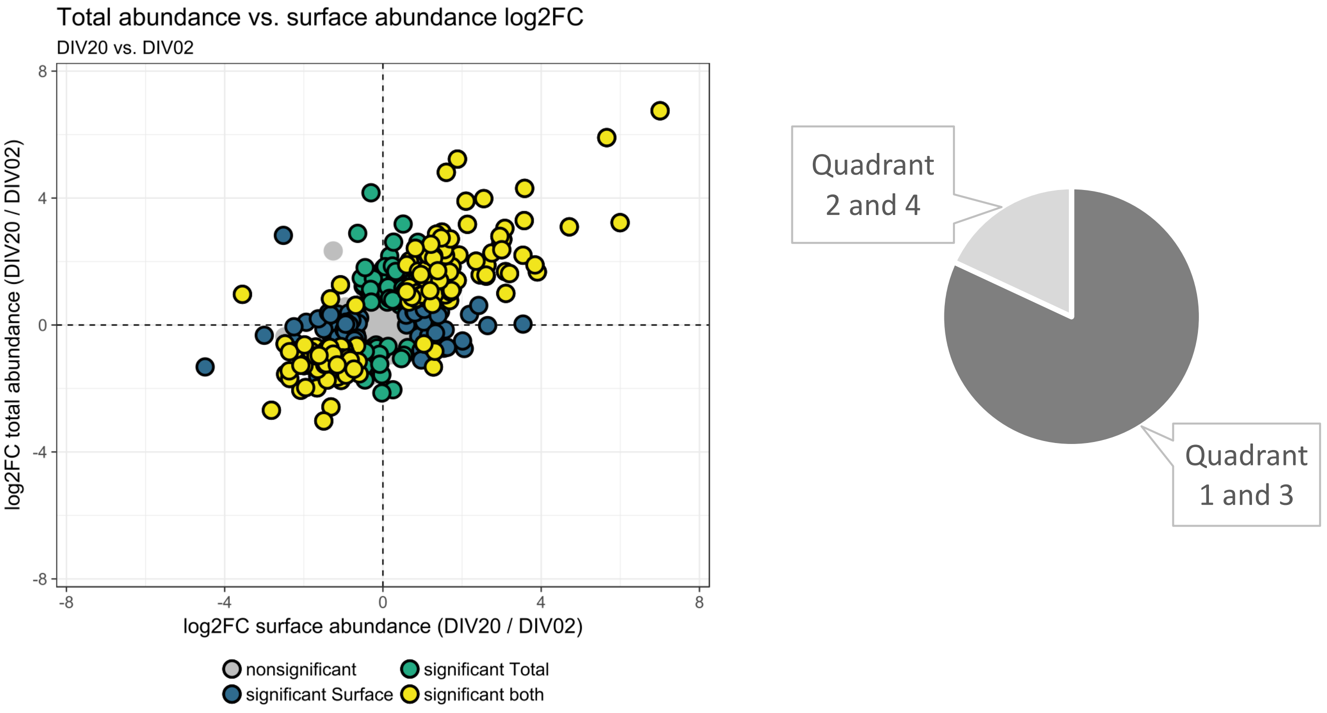

Supplementary Figure 10. Abundance change for surface and total protein pools for the most distant time points. Left: Scatterplot comparing 2 DIV with 20 DIV. Significantly regulated proteins (fold-change > 1.5 and $p < 0.05$) are indicated by color. Right: Pie chart of protein counts present in quadrants 2 and 4 (18%) and 1 and 3 (82%).

Supplementary Figure 11

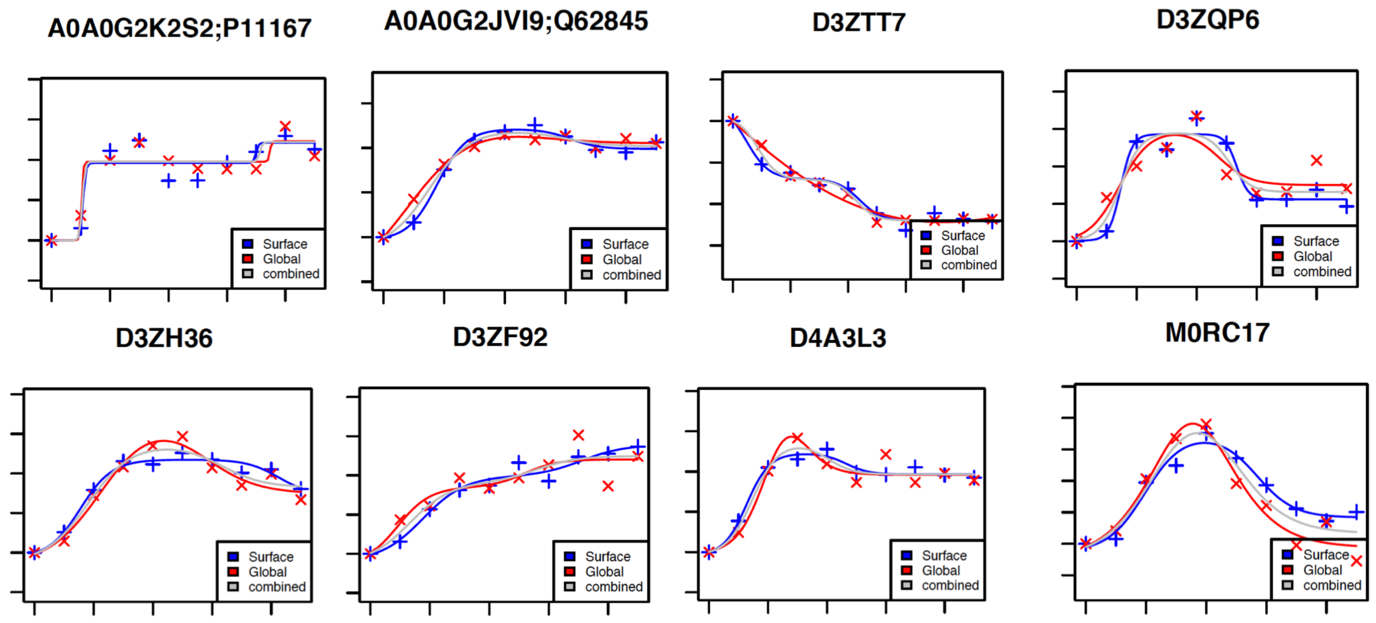

Supplementary Figure 11. Impulse model fit for data from a selection of surface proteins with overlapping surface and total abundance profiles. Crosses indicate normalized median abundance values of three replicates for each time point for surface (blue) and total (red) pools. Lines indicate impulse model fits; grey lines are fits to combined data from surface and total pool analyses[50].

Supplementary Figure 12

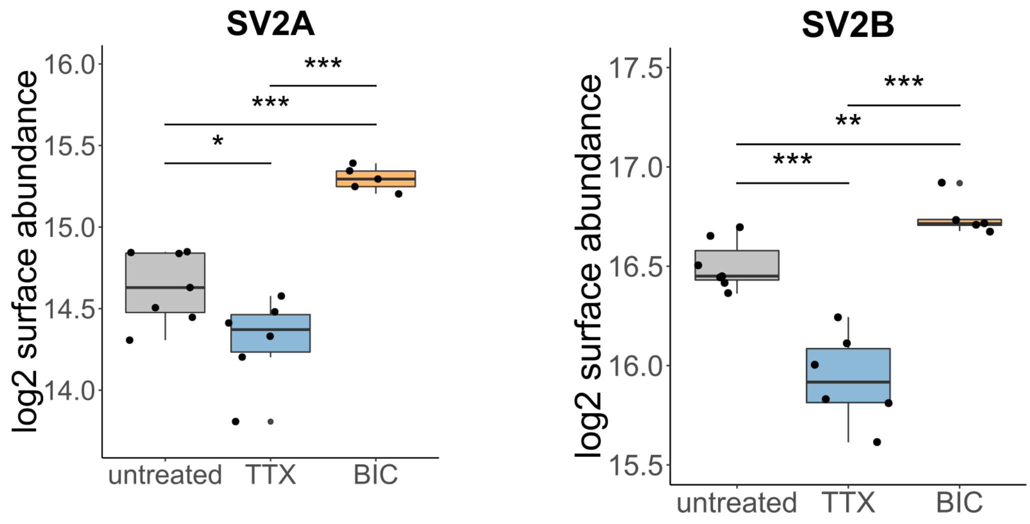

Supplementary Figure 12. Boxplots showing distributions of log2 surface abundances of **b**) SV2a and **c**) SV2b for replicates of untreated (n=7), TTX (n=6) and BIC (n=5). Boxes indicate median and percentiles (25th and 75th). *p < 0.05, **p < 0.01, ***p < 0.001, t-test.

Supplementary Figure 13

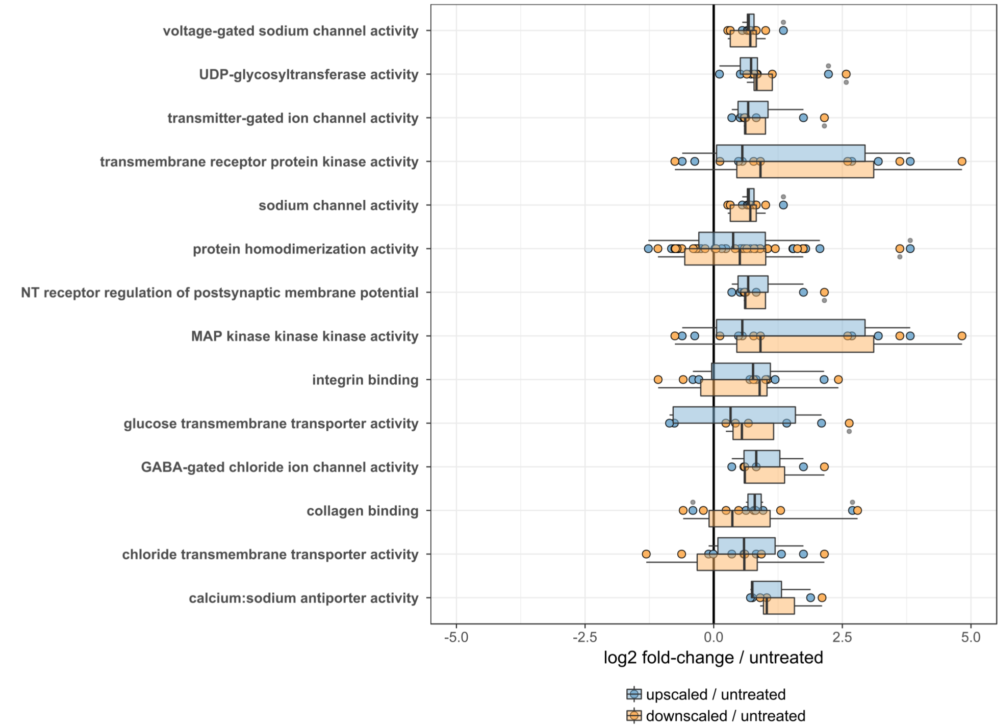

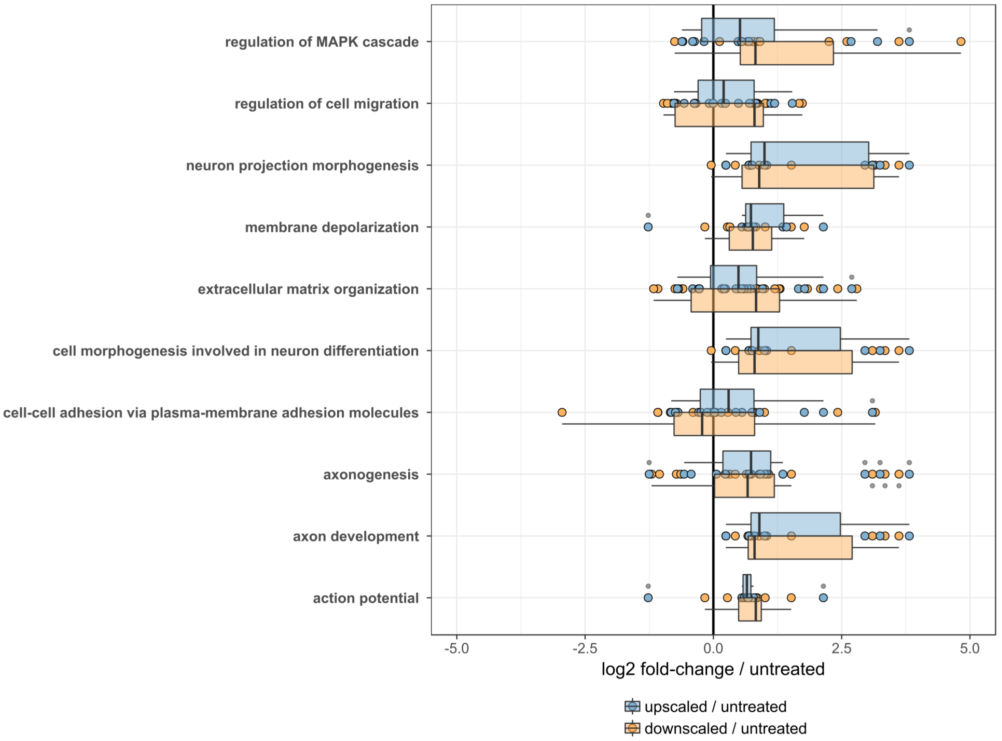

Supplementary Figure 13. GO terms enriched for modulated surface proteins during homeostatic scaling. Proteins mapping to enriched molecular function (top) and biological processes (bottom) GO terms. Boxes indicate median and percentiles (25th and 75th) ($p<0.05$, Fisher's exact test).

Supplementary Figure 14

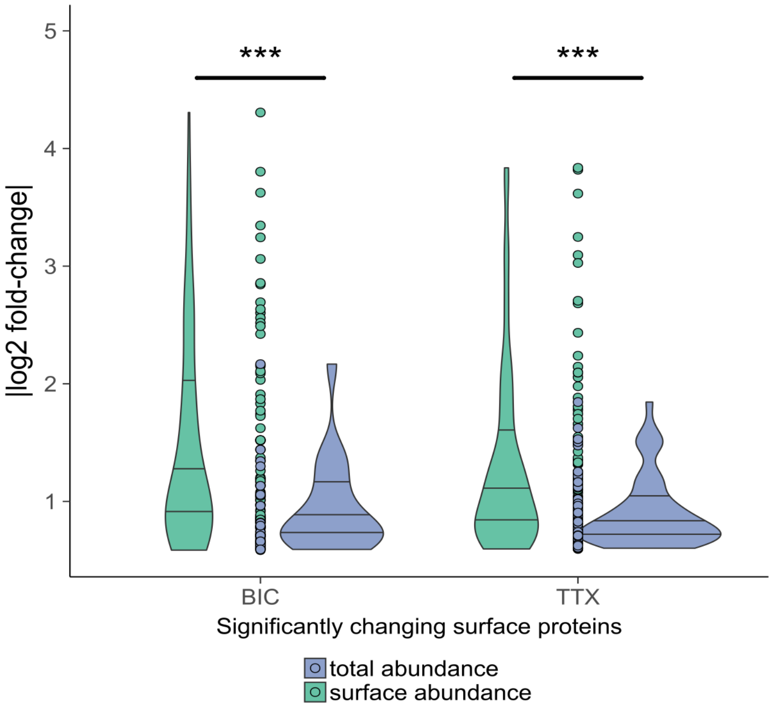

Supplementary Figure 14. Surface pool responds with higher fold-change differences to synaptic scaling than the total protein pool. Violin plots of distributions of absolute fold-change differences (scaling vs. control) for significantly different proteins (fold-change>1.5 and p<0.05) when surface and total abundances per condition were compared. Circles display underlying data points. Lines in violin represent median and percentiles (25th and 75th). ***p < 0.001, t-test.

Supplementary Figure 15

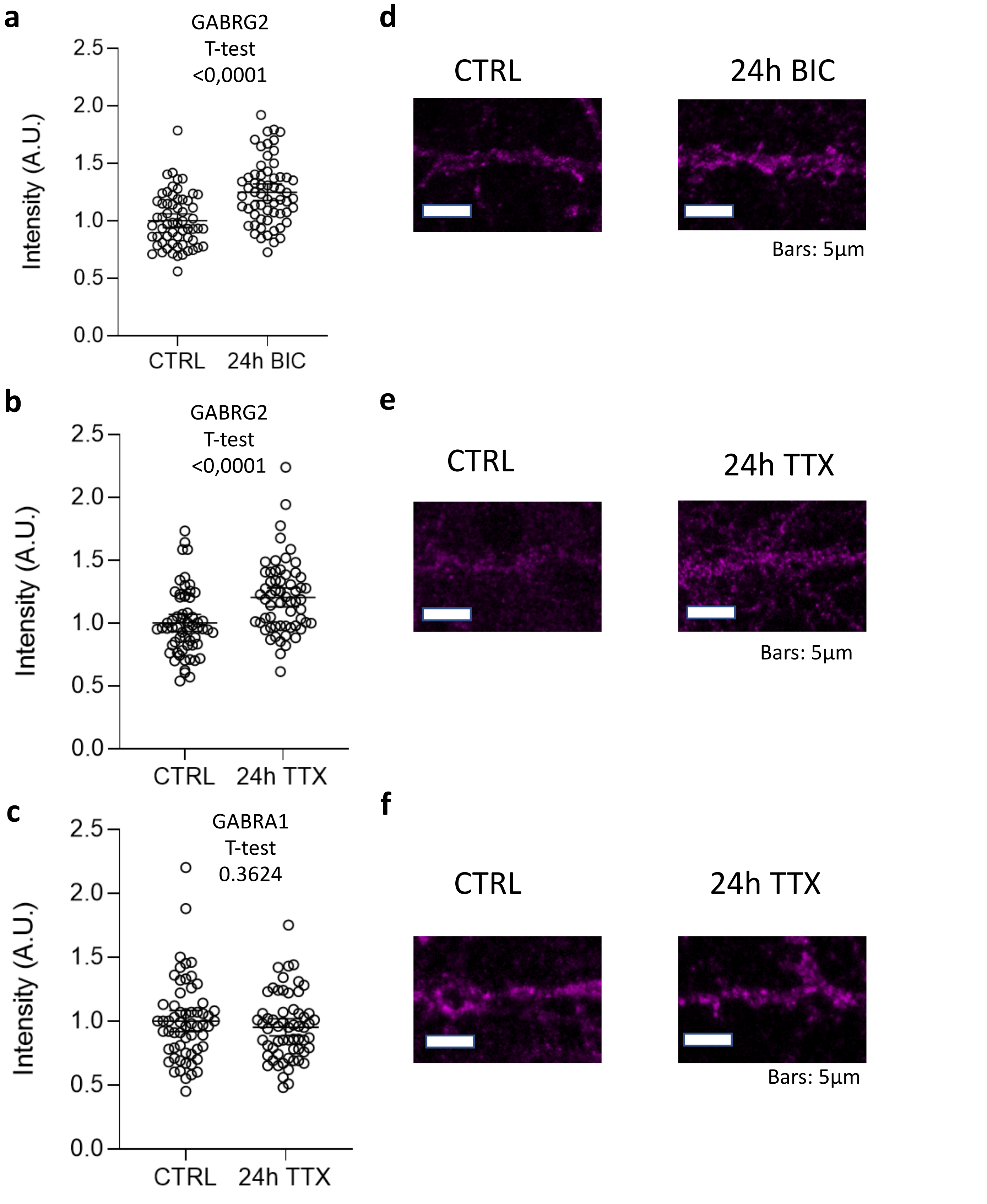

Supplementary Figure 15. a Quantification of mean fluorescent intensity on the primary dendrite surface using antibodies against GABRG2 for synaptic down- (**a**) and upscaling (**b**). n = 60 (CTRL), n = 60 (BIC), n = 60 (TTX). **c** Quantification of mean fluorescent intensity of antibody signal on the primary dendrite surface using antibodies against GABRA1 for synaptic upscaling. n = 60 (CTRL), n = 60 (TTX). Representative images of analysed primary dendrites stained for GABRG2 for synaptic down (**d**) and upscaling (**e**) conditions and for GABRA1 for synaptic upscaling (**f**). Bars 5 µm. Means + 95% CI presented.

Supplementary Figure 16

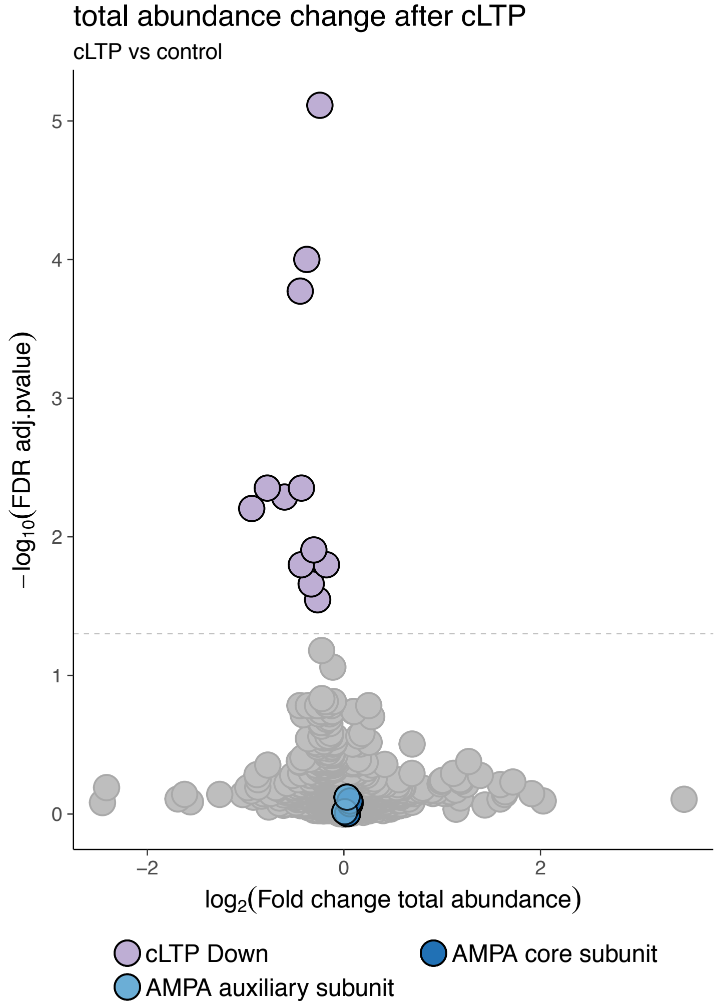

Supplementary Figure 16. Total proteotype analysis after cLTP. Volcano plot of statistical significance (y-axis) vs. surface abundance change (x-axis). Horizontal line is located at adjusted p-value of 0.05.

Supplementary Figure 17

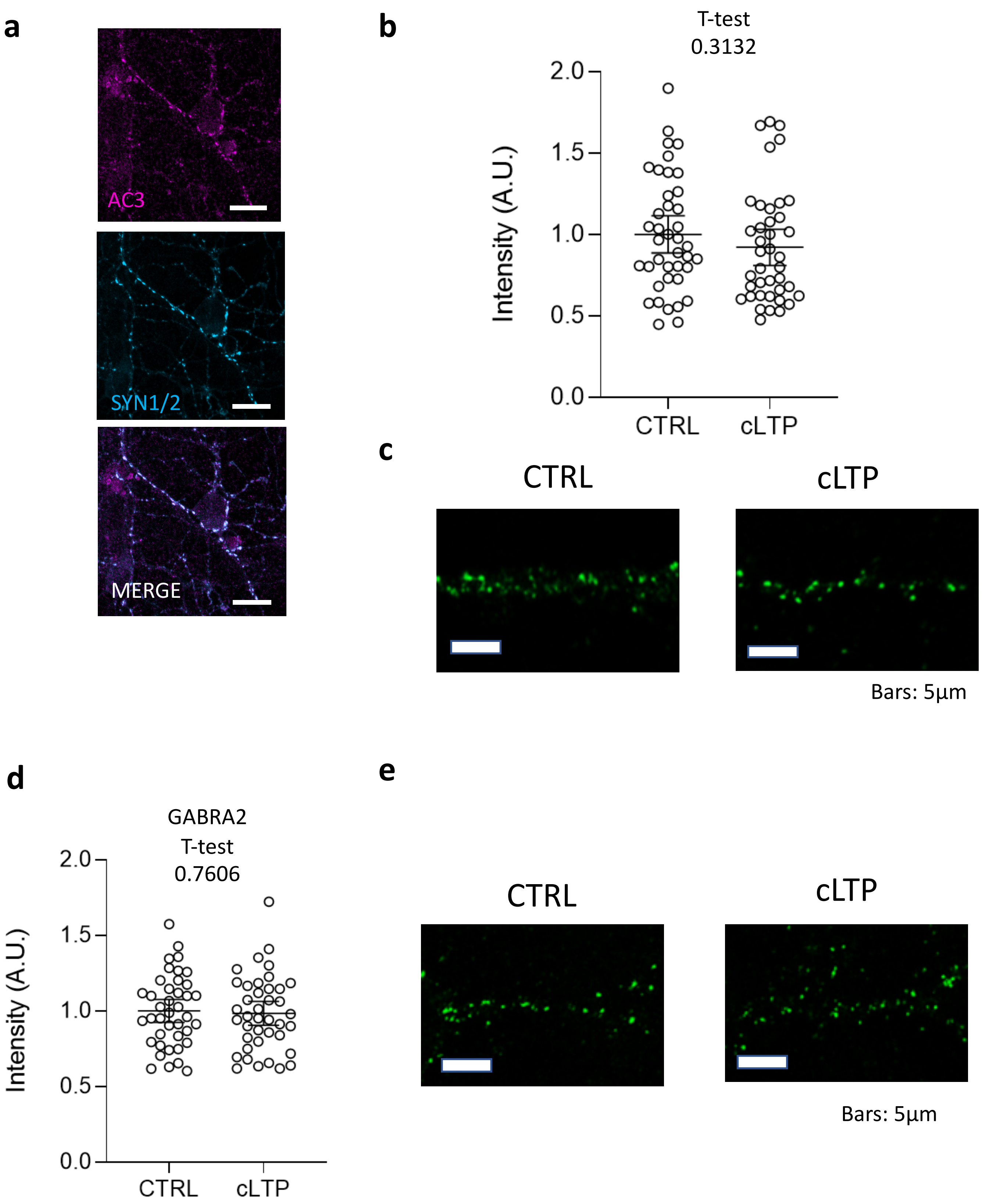

Supplementary Figure 17. Adcy3 localizes to synaptic sites. a Adcy3 immunoreactivity localises to synaptic sites identified using antibodies reactive for the presynaptic marker synapsin 1/2 (Syn 1/2). Bars 20 µm. **b** Quantification of mean fluorescent intensity of antibody signal on the primary dendrite surface using antibodies against GABRA1. n = 60 for both CTRL and cLTP. **c** Representative images of analysed primary dendrites stained for GABRA1 for control and cLTP conditions. Bars 5 µm. **d** Quantification of mean fluorescent intensity on the primary dendrite surface using antibodies against GABRA2. n = 60 for both CTRL and cLTP. **e** Representative images of analysed primary dendrites stained for GABRA2 for control and cLTP conditions. Bars 5 µm. Means + 95% CI presented
