# Supplementary material for: Surfaceome dynamics reveal proteostasis-independent reorganization of neuronal surface proteins during development and synaptic plasticity
Source: Nat Commun. 2020 Oct 5;11:4990. doi: 10.1038/s41467-020-18494-6 (PMC7536423; doi:10.1038/s41467-020-18494-6)
Supplement: Supplementary file 3 — Description of Additional Supplementary Files [file 41467_2020_18494_MOESM3_ESM.docx]

Supplementary data legends

| **Supplementary Data 1: autoCSC quantification during neuronal development in cortical cultures.** |
| --- |
| Sheet 1 (Summary) |
| Sheet 2 (Glycosite analysis): Result of glycosylation site analysis indicate whether the site is annotated in uniprot. |
| Sheet 3 (GroupQuantification): Cell surface abundance values of autoCSC quantified N-glycoproteins per group. |
| Sheet 4 (SampleQuantification): Cell surface abundance values of autoCSC quantified N-glycoproteins per sample. |
| Sheet 5 (Cluster analysis): Results of fuzzy clustering using Mfuzz R package. |
| Sheets 6-15: Result from significance testing of neighbouring time points autoCSC.   \| **Supplementary Data 2**: Proteotype quantification during neuronal development in cortical cultures and comparison with autoCSC. \| \| --- \| \| Sheet 1 (Summary) \| \| Sheet 2 (Total GroupQuantification): Total abundance values (log2) per group. \| \| Sheet 3 (Total SampleQuantification): Total abundance values (log2) per sample. \| \| Sheet 4 (Surface sign test result): Result from significance testing of all time points autoCSC data. \| \| Sheet 5 (Total sign test result): Result from significance testing of all time points total proteotype data. \| \| Sheet 6 (Matching table): Surface and total abundance (log2) matching protein groups including GroupQuantification values. \| \| Sheet 7 (Protein profiles correlation): Correlation of surface and total abundance protein profiles . \| \| Sheet 8 (FC correlation): Correlation of surface and total abundance fold-change differences for pair-wise comparisons of all time points. \| \| Sheet 9 (Overlap sign. regulated): Overlap of significantly regulated proteins of pair-wise comparisons of all time points. \| \| Sheet 10 (ImpulseDE results): Results from ImpulseDE significance testing of surface and total abundance time series. Includes 128 significantly different proteins.  Sheet 11 (ImpulseDE fit): Parameter sets for model fits from ImpulseDE.   \| **Supplementary Data 3**: autoCSC and proteotype analysis for homeostatic plasticity. \| \| --- \| \| Sheet 1 (Summary) \| \| Sheet 2 (HP Surface GroupQuant): Homeostatic plasticity, log2 cell surface abundance values of autoCSC quantified N-glycoproteins per group \| \| Sheet 3 (HP Surface SampleQuant): Homeostatic plasticity, log2 cell surface abundance values of autoCSC quantified N-glycoproteins per sample. \| \| Sheet 3 (HP Surface sign testing): Homeostatic plasticity, results from significance testing autoCSC. \| \| Sheet 4 (HP Total GroupQuant): Homeostatic plasticity, log2 total abundance values per group \| \| Sheet 5 (HP Total SampleQuant): Homeostatic plasticity, log2 total abundance values per sample. \| \| Sheet 6 (HP Total sign testing): Homeostatic plasticity, results from significance testing proteotype analysis. \| \| \| \| **Supplementary Data 4:** autoCSC and proteotype analysis for cLTP. \| \| --- \| \| Sheet 1 (Summary) \| \| Sheet 2 (cLTP Surface GroupQuant): cLTP, log2 cell surface abundance values of autoCSC quantified N-glycoproteins per group \| \| Sheet 3 (cLTP Surface SampleQuant): cLTP, log2 cell surface abundance values of autoCSC quantified N-glycoproteins per sample. \| \| Sheet 3 (cLTP Surface sign testing): cLTP, results from significance testing autoCSC. \| \| Sheet 4 (cLTP Total GroupQuant): cLTP, log2 total abundance values per group \| \| Sheet 5 (cLTP Total SampleQuant): cLTP, log2 total abundance values per sample. \| \| Sheet 6 (cLTP Total sign testing): cLTP, results from significance testing proteotype analysis. \| \| |
