# Supplementary material for: Surfaceome dynamics reveal proteostasis-independent reorganization of neuronal surface proteins during development and synaptic plasticity
Source: Nat Commun. 2020 Oct 5;11:4990. doi: 10.1038/s41467-020-18494-6 (PMC7536423; doi:10.1038/s41467-020-18494-6)
Supplement: Supplementary file 8 — Reporting Summary [file 41467_2020_18494_MOESM8_ESM.pdf]

nature research

Corresponding author(s): Bernd Wollscheid

Last updated by author(s): 12.04.2020

Reporting Summary

Nature Research wishes to improve the reproducibility of the work that we publish. This form provides structure for consistency and transparency in reporting. For further information on Nature Research policies, see Authors & Referees and the Editorial Policy Checklist .

Statistics

For all statistical analyses, confirm that the following items are present in the figure legend, table legend, main text, or Methods section.

n/a	Confirmed	
☐	☒	The exact sample size (*n*) for each experimental group/condition, given as a discrete number and unit of measurement
☐	☒	A statement on whether measurements were taken from distinct samples or whether the same sample was measured repeatedly
☐	☒	The statistical test(s) used AND whether they are one- or two-sided<br>*Only common tests should be described solely by name; describe more complex techniques in the Methods section.*
☒	☐	A description of all covariates tested
☐	☒	A description of any assumptions or corrections, such as tests of normality and adjustment for multiple comparisons
☐	☒	A full description of the statistical parameters including central tendency (e.g. means) or other basic estimates (e.g. regression coefficient) AND variation (e.g. standard deviation) or associated estimates of uncertainty (e.g. confidence intervals)
☒	☐	For null hypothesis testing, the test statistic (e.g. *F*, *t*, *r*) with confidence intervals, effect sizes, degrees of freedom and *P* value noted<br>*Give P values as exact values whenever suitable.*
☒	☐	For Bayesian analysis, information on the choice of priors and Markov chain Monte Carlo settings
☒	☐	For hierarchical and complex designs, identification of the appropriate level for tests and full reporting of outcomes
☒	☐	Estimates of effect sizes (e.g. Cohen's *d*, Pearson's *r*), indicating how they were calculated

Our web collection on statistics for biologists contains articles on many of the points above.

Software and code

Policy information about availability of computer code

Data collection	Mass spectra were acquired using a Thermo Fisher Scientific mass spectrometers operated with the vendor provided Tune and Xcalibur software. Images were acquired using a Zeiss LSM upright laser scanning confocal microscope and Zen software.
Data analysis	Acquired spectra containing raw files were processed with either Proteome Discoverer (v.2.1, Thermo Scientific) using SEQUEST search engine and/or Spectronaut (v.12, Biognosys AG). Bioinformatic analysis was performed in python (v.3.6) and the R statistical computing environment (v.3.4.0) using the R package MSstats (v.3.8.6), mfuzz (v.2.36.0), topGO (v.2.28.0) and ImpulseDE (1.2.0). Images were analyzed using ImageJ and the Synapse Counter plugin with default settings. The electrophysiology data analysis was performed using Clampfit (Molecular Devices).

For manuscripts utilizing custom algorithms or software that are central to the research but not yet described in published literature, software must be made available to editors/reviewers. We strongly encourage code deposition in a community repository (e.g. GitHub). See the Nature Research guidelines for submitting code & software for further information.

Data

Policy information about availability of data

All manuscripts must include a data availability statement. This statement should provide the following information, where applicable:

- Accession codes, unique identifiers, or web links for publicly available datasets
- A list of figures that have associated raw data
- A description of any restrictions on data availability

The mass spectrometry proteomics data have been deposited to the ProteomeXchange Consortium via the PRIDE partner repository with the dataset identifier PXD014790. Selected data is available at neurosurfaceome.ethz.ch.

Field-specific reporting

Please select the one below that is the best fit for your research. If you are not sure, read the appropriate sections before making your selection.

☒ Life sciences ☐ Behavioural & social sciences ☐ Ecological, evolutionary & environmental sciences

For a reference copy of the document with all sections, see nature.com/documents/nr-reporting-summary-flat.pdf

Life sciences study design

All studies must disclose on these points even when the disclosure is negative.

Sample size	Sample size was not predetermined by statistical methods for relative quantitative mass spectrometry experiments. However, for each protein, quantitative values were modeled based on the intensity of at least two features (peptide fragments) per proteotypic peptide using the Tukey's median polish method to ensure robust label-free quantification suitable for statistical testing.
Data exclusions	Peptide identifications mapping to decoy or contaminant proteins or internal reference peptides were excluded from further analysis. Additionally, peptides were filtered based on MS/MS identification score to ensure a false discovery rate of < 1%. Outliers based on hierarchical clustering were removed when indicated in the methods. For electrophysiology, cells without any detected fast EPSC were not considered in the amplitude comparisons.
Replication	Automated MS-based Cell Surface Capture (autoCSC) was used to track quantitative changes of the cell surface during neuronal development and synaptic plasticity. Surface expression of selected candidates was assessed using antibody-based neuro-morphology analysis and replicated the results from autoCSC.
Randomization	Samples were block-randomized for mass spectrometry aquistion.
Blinding	Investigators were not blinded to allocation of biological samples. The results of the mass spectrometry experiments are of technical nature and not prone to a potential observer bias. Electrophysiology experiments were performed blinded.

Reporting for specific materials, systems and methods

We require information from authors about some types of materials, experimental systems and methods used in many studies. Here, indicate whether each material, system or method listed is relevant to your study. If you are not sure if a list item applies to your research, read the appropriate section before selecting a response.

Materials & experimental systems

n/a	Involved in the study	
☐	☒	Antibodies
☒	☐	Eukaryotic cell lines
☒	☐	Palaeontology
☐	☒	Animals and other organisms
☒	☐	Human research participants
☒	☐	Clinical data

Methods

n/a	Involved in the study	
☒	☐	ChIP-seq
☒	☐	Flow cytometry
☒	☐	MRI-based neuroimaging

Antibodies

Antibodies used	The following antibodies were used for immunocytochemistry: PSD95 (mouse monoclonal, clone 6G6-1C9, Thermo Fisher Scientific, 1:1000), gephyrin (recombinant rabbit purified IgG, clone RbmAb7a, Synaptic Systems, 1:500), synapsin (monoclonal mouse IgG fluorescently labeled with Oyster 488, clone 46.1, Synaptic Systems, 1:1000). ADGRB1 (ABR-021, Alomone, 1:100), AMPARs (182 411, Synaptic Systems, 1:100), AC3 (AAR-043, Alomone, 1:100), map2 (rabbit polyclonal, ab32454, abcam, 1:500) and GFAP (mouse monoclonal, Cell Signalling 3670, 1:500). Previously characterized homemade antibodies targeting the extracellular domain of GABAA receptors (guineapig anti-GABRG2, guineapig anti-GABRA1, guineapig anti-GABRA2) were diluted at 1:400.
Validation	Commercial antibodies were quality control tested by immunofluorescent staining with immunocytochemistry analysis by the manufacturer.

Animals and other organisms

Policy information about studies involving animals; ARRIVE guidelines recommended for reporting animal research

Laboratory animals	Species: Rattus Norvegicus; Strain: Sprague Dawley. Age P0/1; used for primary neuron culture. Gender: Males and females.
Wild animals	The study did not involve wild animals.
Field-collected samples	The study did not involve samples collected from the field.
Ethics oversight	All animal experiments were carried out under institutional guidelines (ZH172/18 Kanton Zürich Gesundheitsdirektion Veterinäramt)

Note that full information on the approval of the study protocol must also be provided in the manuscript.
